# High Levels of Cyclic Diguanylate Interfere with Beneficial Bacterial Colonization

**DOI:** 10.1128/mbio.01671-22

**Published:** 2022-08-02

**Authors:** Ruth Y. Isenberg, David G. Christensen, Karen L. Visick, Mark J. Mandel

**Affiliations:** a Department of Medical Microbiology and Immunology, University of Wisconsin—Madison, Madison, Wisconsin, USA; b Microbiology Doctoral Training Program, University of Wisconsin—Madison, Madison, Wisconsin, USA; c Department of Microbiology and Immunology, Loyola University Stritch School of Medicine, Maywood, Illinois, USA; Max Planck Institute for Marine Microbiology

**Keywords:** *Vibrio fischeri*, microbiome, *Euprymna scolopes*, squid, c-di-GMP, biofilm, flagellar motility, Hawaiian bobtail squid, bacterial colonization, cyclic diguanylate, motility, symbiosis

## Abstract

During colonization of the Hawaiian bobtail squid (*Euprymna scolopes*), Vibrio fischeri bacteria undergo a lifestyle transition from a planktonic motile state in the environment to a biofilm state in host mucus. Cyclic diguanylate (c-di-GMP) is a cytoplasmic signaling molecule that is important for regulating motility-biofilm transitions in many bacterial species. V. fischeri encodes 50 proteins predicted to synthesize and/or degrade c-di-GMP, but a role for c-di-GMP regulation during host colonization has not been investigated. We examined strains exhibiting either low or high levels of c-di-GMP during squid colonization and found that while a low-c-di-GMP strain had no colonization defect, a high c-di-GMP strain was severely impaired. Expression of a heterologous c-di-GMP phosphodiesterase restored colonization, demonstrating that the effect is due to high c-di-GMP levels. In the constitutive high-c-di-GMP state, colonizing V. fischeri exhibited reduced motility, altered biofilm aggregate morphology, and a regulatory interaction where transcription of one polysaccharide locus is inhibited by the presence of the other polysaccharide. Our results highlight the importance of proper c-di-GMP regulation during beneficial animal colonization, illustrate multiple pathways regulated by c-di-GMP in the host, and uncover an interplay of multiple exopolysaccharide systems in host-associated aggregates.

## INTRODUCTION

In both pathogenic and beneficial associations, bacteria transition from a motile, planktonic state in the environment to a surface-associated biofilm state within the host. Cyclic diguanylate (c-di-GMP) is an intracellular signaling molecule that regulates this lifestyle transition for many bacterial species, including Vibrio cholerae, Pseudomonas aeruginosa, Caulobacter crescentus, and Agrobacterium tumefaciens (1, 2). Generally, higher intracellular c-di-GMP concentrations lead to increased biofilm formation and decreased motility, while lower intracellular c-di-GMP concentrations lead to the opposite phenotypes. c-di-GMP levels are regulated by diguanylate cyclase (DGC) proteins, which synthesize c-di-GMP, and phosphodiesterase (PDE) proteins, which degrade c-di-GMP. DGCs contain a GGDEF domain and PDEs contain either an EAL or HD-GYP domain. While the effects of c-di-GMP on motility, biofilm formation, and infectivity of pathogens such as V. cholerae have been extensively studied (3), the impact of c-di-GMP during the establishment of beneficial bacterial symbioses is not well understood. The focus of this study is to examine how c-di-GMP impacts beneficial colonization in a system where we can manipulate intracellular levels of the compound and dissect the colonization process in detail.

Vibrio fischeri is the exclusive, beneficial light organ symbiont of the Hawaiian bobtail squid (*Euprymna scolopes*). V. fischeri undergoes the motility-to-biofilm lifestyle transition during the initiation of host colonization (4–7). While V. fischeri is solitary and expresses polar flagella in seawater, upon encountering the juvenile bobtail squid light organ the bacteria require the symbiosis polysaccharide (Syp) to aggregate in the host mucus prior to commencing further colonization (5, 8–11). The bacterial aggregates form on the outer face of the light organ independent of flagellar motility and are influenced by physical currents generated by cilia on the light organ surface (4, 12). Host-produced nitric oxide stimulates dispersal from the biofilm aggregates (13), and the bacteria swim through the pore into the light organ ducts using flagellar motility and chemotaxis toward squid-derived chitin oligosaccharides (8, 14–17). The symbionts pass through an antechamber and bottleneck before reaching the crypts of the light organ, where they reach high density and produce light to camouflage the shadow of the squid host (18–20). The initiation of this process—including biofilm formation, chemotaxis, and motility—occurs only when the squid is newly hatched, as colonizing bacteria seed the host for its lifetime. Therefore, the mechanisms that regulate these processes are fundamental to selection for the correct symbionts and the establishment of the association.

As with the pathogens noted above, V. fischeri (strain ES114) encodes a large number of proteins predicted to regulate c-di-GMP levels: 50 total that encompass 28 DGCs, 14 PDEs, 5 DGC/PDEs, and 3 proteins that are predicted to be degenerate (21). Roles for DGCs MifA and MifB, and PDEs BinA and PdeV in V. fischeri motility and/or biofilm formation have been described (22–24). However, those studies did not examine individual mutants during squid colonization, and a large-scale transposon-insertion sequencing study did not identify individual mutations in genes related to c-di-GMP that were predicted to have significant phenotypes in the squid host (25). Given the predicted complexity of the c-di-GMP network in V. fischeri, we used new technology (26) to generate strains lacking multiple DGCs or PDEs to mimic either low or high c-di-GMP levels, and we applied these strains to determine the effects of c-di-GMP extremes on squid colonization. This approach allowed us to directly study the role of c-di-GMP *in vivo* during beneficial colonization, to determine bacterial behaviors that are influenced by the compound during animal colonization, and to reveal signaling interactions that are not evident during growth in culture.

## RESULTS

### Deletion of multiple DGCs or PDEs effectively manipulates c-di-GMP levels in the symbiont *V. fischeri*.

**FIG 1:**
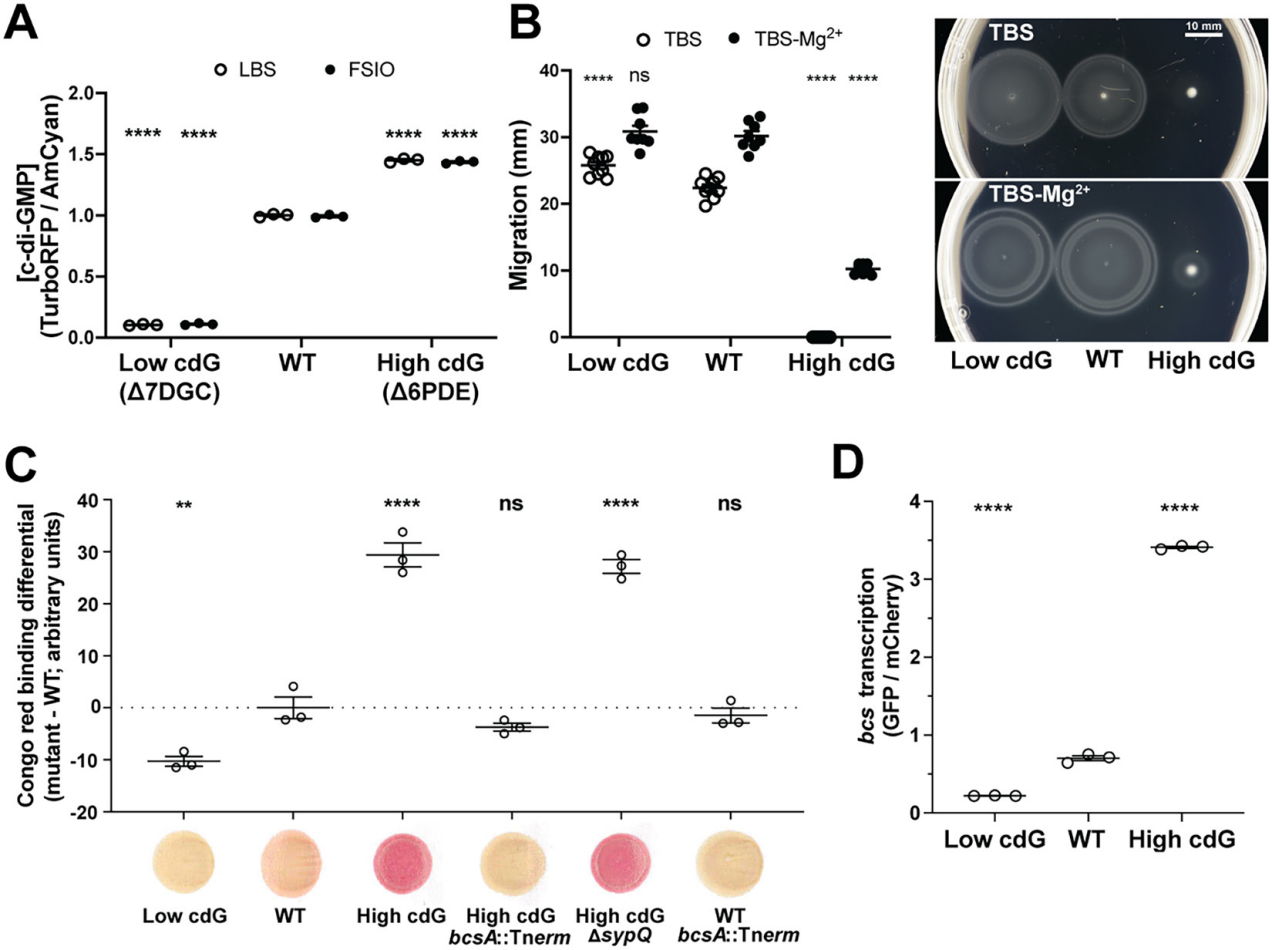
High c-di-GMP levels inhibit swimming motility, promote Congo red binding, and promote cellulose gene transcription. (A) Quantification of c-di-GMP concentration for indicated V. fischeri strains in LBS and FSIO using the pFY4535 c-di-GMP reporter plasmid. Constitutive AmCyan was used to normalize GFP to cell density. For each strain, *n *=* *3 biological and *n *=* *3 technical replicates per biological replicate, each point represents the mean of technical replicates, average bars represent the means of biological replicates, and error bars represent standard errors of the mean. (B) Quantification of migration through soft (0.3%) agar for indicated V. fischeri strains. For each strain, *n *= 8 to 9 biological replicates, average bars represent means, and error bars represent standard errors of the mean. Motility plate images are representative. (C) Quantification of Congo red binding for indicated V. fischeri strains. For each strain, *n *=* *3 technical replicates, average bars represent means, and error bars represent standard errors of the mean. Congo red spot images are representative. (D) Quantification of *in vitro bcs* transcription by indicated V. fischeri strains using the pRYI063 *bcsQ*′-*gfp*^+^ transcriptional reporter plasmid. For each strain, *n* = 3 biological replicates. Points represent each biological replicate, average bars represent the means, and error bars represent standard errors of the mean. Constitutive mCherry was used to normalize GFP to cell density. For panels A to D, one-way analysis of variance (ANOVA) was used for statistical analysis, and asterisks represent significance relative to the WT strain (ns, not significant; *, *P* < 0.05; **, *P* < 0.006; ****, *P* < 0.0001).

To determine the role that c-di-GMP plays during the beneficial colonization of *E. scolopes* by V. fischeri, we constructed strains that were altered in their basal levels of c-di-GMP. While it is not yet feasible to delete all 50 c-di-GMP-related genes in a single strain, we applied recently developed technology that facilitates rapid construction of multiple gene deletions (26) to generate a strain that lacked seven DGCs (Δ7DGC; lacking *VF_1200*, *mifA*, *VF_1245*, *VF_A0342*-*A0343*, *VF_1639*, and *VF_A0216*) and another that lacked six PDEs (Δ6PDE; lacking *pdeV*, *VF_2480*, *binA*, *VF_0087*, *VF_1603*, and *VF_A0506*). These two strains were derived using a recently generated collection of 50 single mutants (27). The specific gene disruptions were chosen based both on technical reasons (e.g., location of a gene of interest in the chromosome or the specific antibiotic resistance cassette present) and on preliminary phenotypic assessments. For the latter, we focused on mutations that, in single mutant experiments, impacted known c-di-GMP-dependent phenotypes, primarily the control over cellulose production (via Congo red binding) (24). Ultimately, our goal was to generate strains with decreased and increased levels of c-di-GMP. To determine whether we achieved this goal, we examined the levels of c-di-GMP in these strains using the pFY4535 c-di-GMP reporter (28) and found that the Δ7DGC strain had c-di-GMP levels >9-fold lower than the parent strain, while the Δ6PDE strain exhibited c-di-GMP levels ~1.5-fold higher (Fig. 1A). These differences were observed in rich medium, as well as in filter-sterilized Instant Ocean (FSIO), under conditions that mimic squid inoculation (Fig. 1A), suggesting to us that these strains would be effective for studying the role of c-di-GMP during squid colonization. We examined phenotypes known to be regulated by c-di-GMP, including cellulose biofilm formation and flagellar motility. The Δ7DGC strain displayed elevated motility on TBS soft agar, reduced Congo red binding, and reduced activity of a *bcsQ′-gfp^+^* cellulose locus transcriptional reporter (Fig. 1B to D). The Δ6PDE strain exhibited the opposite phenotypes (Fig. 1B to D). These data supported our contention that we could use the multiple deletion approach to examine c-di-GMP regulation in the symbiont. Together, these data and those below support that the Δ7DGC strain represents cells in a low-c-di-GMP state, whereas the Δ6PDE cells are in a high-c-di-GMP state, and for the remainder of the study we will refer to these strains as the “Low cdG” and “High cdG” strains, respectively.

### Elevated c-di-GMP levels interfere with productive colonization initiation.

**FIG 2:**
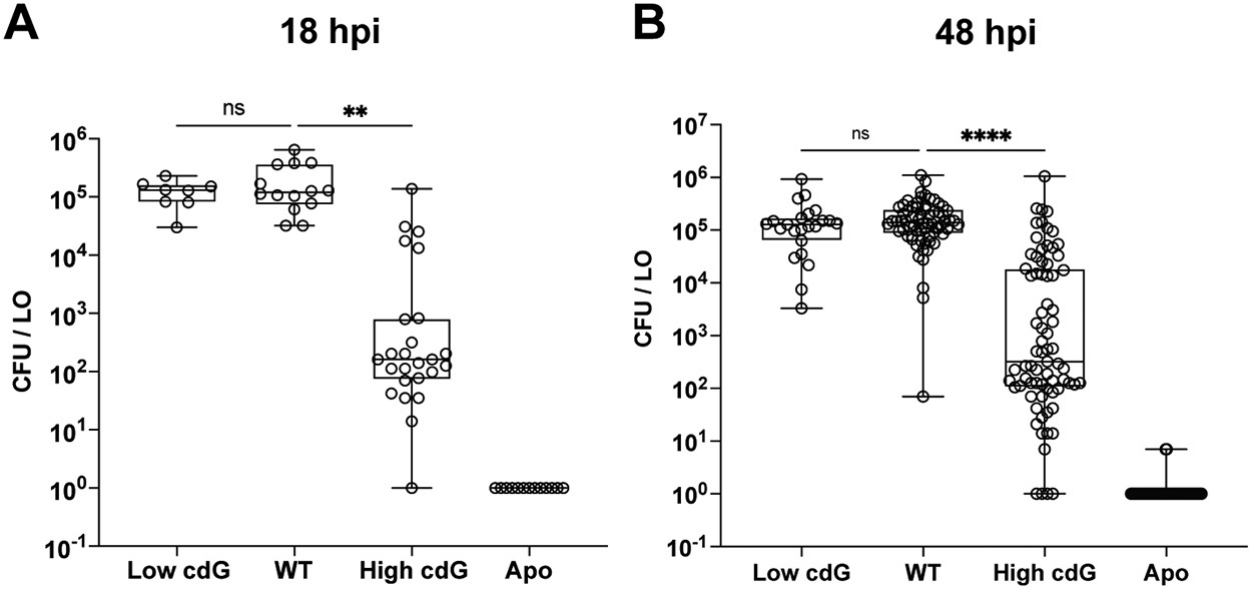
Elevated c-di-GMP levels inhibit host colonization. (A) Quantification of squid colonization levels at 18 hpi by indicated V. fischeri strains and aposymbiotic (Apo) control. Sample sizes from left to right are 8, 14, 25, and 13 squid. (B) Quantification of squid colonization levels at 48 hpi by indicated V. fischeri strains and aposymbiotic (Apo) control. Sample sizes from left to right are 23, 68, 75, and 65 squid. For panels A and B, box-and-whisker plots represent the minimum, 25th percentile, median, 75th percentile, and maximum. A Kruskal-Wallis test was performed for statistical analysis for squid that were introduced to bacteria (ns, not significant; **, *P* < 0.002; ****, *P* < 0.0001).

To determine whether the lower and/or higher c-di-GMP levels impacted symbiotic colonization, we introduced the Low cdG and High cdG strains to newly hatched *E. scolopes* squid. Each strain was individually inoculated into a bowl with hatchling squid and allowed to initiate colonization for 3 h. The squid were transferred to new seawater, and the bacterial load in each host was measured at 18 and 48 h postinoculation (hpi) (29). For wild-type (WT) V. fischeri ES114, this process leads to approximately 10^5^ CFU per squid at 18 and 48 hpi, and a similar yield was observed for the Low cdG strain (Fig. 2). This was surprising to us: given that there are 50 genes that encode c-di-GMP synthesis/degradation factors, we expected the compound to play an important role during colonization, yet downregulation of c-di-GMP levels by >9-fold did not impact the ability to colonize the squid host (Fig. 2). To determine whether the Low cdG strain exhibited a more subtle colonization defect that we were not detecting in this single strain colonization assay, we performed a competitive colonization assay in which the Low cdG and WT strains were coinoculated. We reasoned that if the Low cdG strain had a slight defect in the host, then the competitive assay could reveal this effect. However, in the competitive assay, the Low cdG strain exhibited similar fitness as the WT strain (see Fig. S1 in the supplemental material). In contrast, in the single-strain colonization assay, the High cdG strain had a substantial deficit in bacterial yield in the host as early as 18 hpi and also at 48 hpi, with a median CFU below 10^3^ per light organ (Fig. 2). In the competitive colonization, the High cdG strain displayed a significant deficit, since WT outcompeted the High cdG strain by >3,000-fold (see Fig. S1). Growth of the High cdG strain in culture was similar to that of the WT (see Fig. S2), arguing that the colonization defect is due to a developmental deficit in the host and not an inherent growth property of the strain. From these data, we conclude that high levels of c-di-GMP are detrimental for the initiation of a beneficial colonization in the squid light organ, whereas low levels of c-di-GMP do not impact the colonization behavior during the first 48 h of symbiosis.

10.1128/mbio.01671-22.1 FIG S1 Low levels of c-di-GMP do not inhibit host colonization. Quantification of squid competitive colonization index at 48 hpi by indicated V. fischeri strains. Competitive index represents the log_10_[(test strain/WT)_output_/(test strain/WT)_input_]. Box-and-whisker plots represent the minimum, 25th percentile, median, 75th percentile, and maximum. Sample sizes from left to right are 36, 26, and 20 squid. A Kruskal-Wallis test was performed for statistical analysis (ns, not significant; ****, *P* < 0.0001). Download FIG S1, PDF file, 0.05 MB. Copyright © 2022 Isenberg et al. 2022 Isenberg et al. https://creativecommons.org/licenses/by/4.0/ This content is distributed under the terms of the Creative Commons Attribution 4.0 International license.

10.1128/mbio.01671-22.2 FIG S2 The High c-di-GMP V. fischeri strain does not have a growth defect. (A) Growth curves for V. fischeri and indicated mutants. For each strain, *n *=* *3 biological and *n *=* *6 technical replicates per biological replicate. Points represent the means of technical replicates. Error bars represent standard errors of the mean. (B) Doubling times for V. fischeri and indicated mutants. Average bars represent the means of biological replicates. Points represent the means of technical replicates. Error bars represent standard errors of the mean. One-way ANOVA was used for statistical analysis (ns, not significant). The doubling time was calculated using a nonlinear regression analysis of each strain during exponential growth phase. For panels A and B, outliers were excluded from analysis. Download FIG S2, PDF file, 0.1 MB. Copyright © 2022 Isenberg et al. 2022 Isenberg et al. https://creativecommons.org/licenses/by/4.0/ This content is distributed under the terms of the Creative Commons Attribution 4.0 International license.

**FIG 3:**
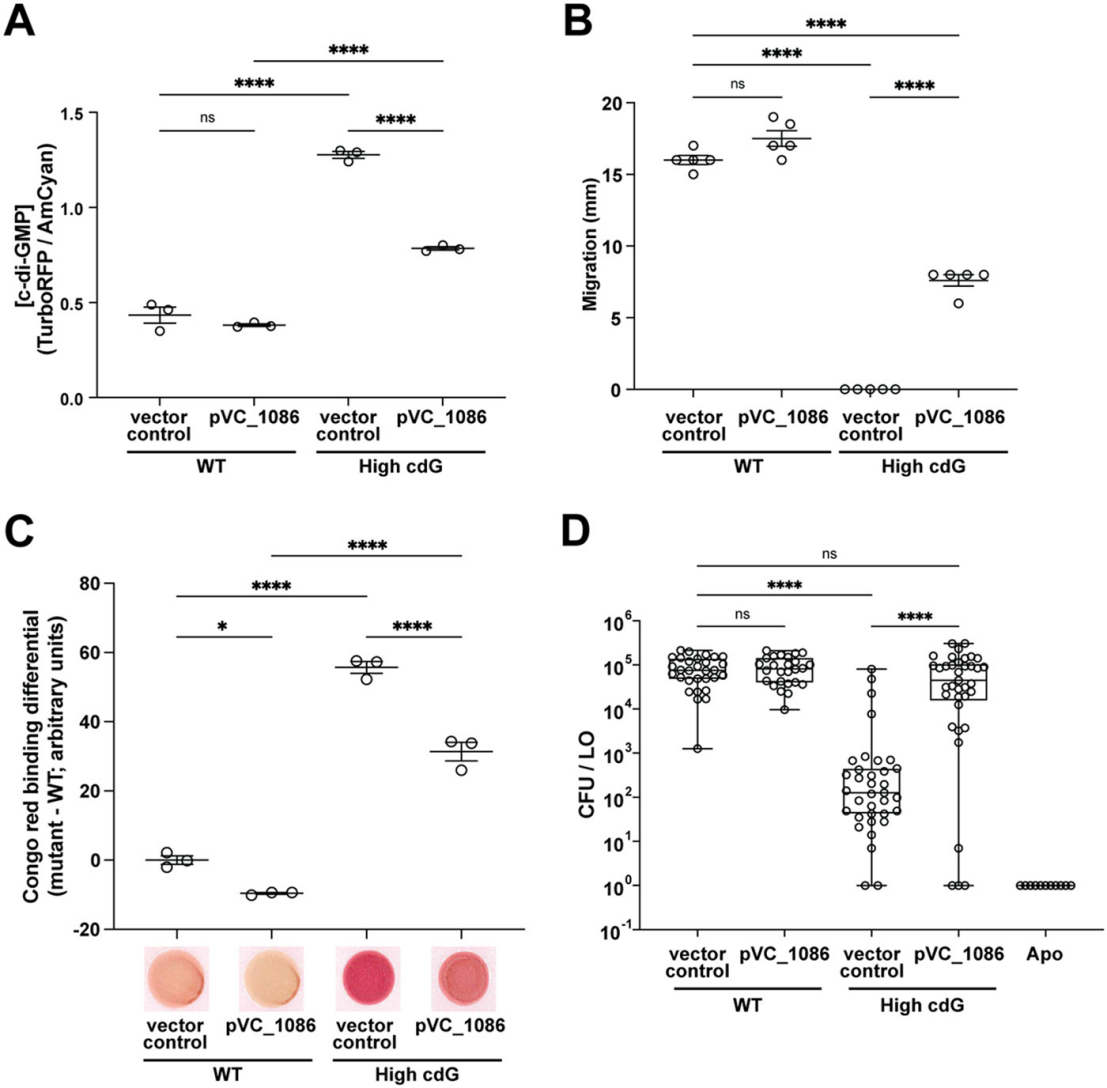
Reduced swimming motility, increased Congo red binding, and diminished squid colonization of the High cdG strain are dependent on high c-di-GMP levels. (A) Quantification of c-di-GMP concentration for indicated V. fischeri strains using the pFY4535 c-di-GMP reporter plasmid. For each strain, *n *=* *3 biological and *n *=* *3 technical replicates, each dot represents the mean of technical replicates, average bars represent the means of biological replicates, and error bars represent standard errors of the mean. (B) Quantification of migration through TBS soft (0.3%) agar for indicated V. fischeri strains. For each strain, *n *=* *5 biological replicates, average bars represent means, and error bars represent standard errors of the mean. (C) Quantification of Congo red binding for indicated V. fischeri strains. For each strain, *n *=* *3 technical replicates, average bars represent the means, and error bars represent standard errors of the mean. Congo red spot images are representative. For panels A to C, one-way ANOVA was used for statistical analysis (ns, not significant; *, *P* = 0.017; ****, *P* < 0.0001). (D) Quantification of squid colonization levels at 48 hpi by indicated V. fischeri strains and aposymbiotic (Apo) control. Box-and-whisker plots represent the minimum, 25th percentile, median, 75th percentile, and maximum. Sample sizes from left to right are 30, 26, 34, 37, and 11 squid. A Kruskal-Wallis test was performed for statistical analysis for squid that were introduced to bacteria (ns, not significant; ****, *P* < 0.0001).

To determine whether the phenotypes we observed in the High cdG strain were due to regulation of c-di-GMP levels or whether they were due to a specific gene product that is absent (i.e., signaling specificity), we introduced a PDE from V. cholerae, VC1086, that is known to be active in heterologous bacteria (30, 31) and that we predicted would lower c-di-GMP levels in V. fischeri. Across the phenotypes tested, the plasmid-expressed VC1086 had only a slight effect in a WT background (Fig. 3). However, in the High cdG background, expression of VC1086 reduced cdG levels by 1.6-fold, restored swimming motility on TBS agar, and reduced Congo red binding 1.7-fold in the High cdG background (Fig. 3A to C). In a squid colonization assay, expression of the V. cholerae PDE rescued colonization of the High cdG strain (Fig. 3D). This set of experiments confirmed that our results were due to altering levels of c-di-GMP rather than the absence of a single gene product. We conclude that lower levels of c-di-GMP are not harmful to establishing a symbiotic association, while higher levels are detrimental to symbiotic initiation.

### The High c-di-GMP strain exhibits flagellar motility *in vivo*.

**FIG 4:**
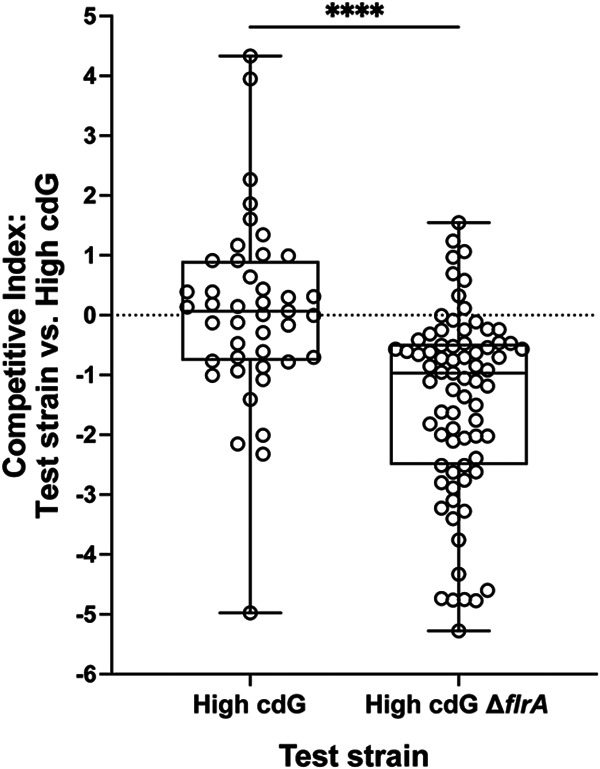
High c-di-GMP levels do not eliminate motility during host colonization. Quantification of squid competitive colonization index at 48 hpi by indicated V. fischeri strains. The competitive index represents the log_10_[(test strain/High cdG)_output_/(test strain/High cdG)_input_]. Box-and-whisker plots represent the minimum, 25th percentile, median, 75th percentile, and maximum. Sample sizes from left to right are 43 and 75 squid. A Kruskal-Wallis test was performed for statistical analysis (****, *P* < 0.0001).

In culture, the High cdG strain had a substantial swimming motility defect (Fig. 1B). We therefore considered the hypothesis that a key basis for the poor colonization by the High cdG strain was due to the altered flagellar motility, which is required for host colonization (8, 15, 17). In culture, we did observe a migration ring in the presence of magnesium sulfate, which promotes V. fischeri motility (32), demonstrating that the strain is not amotile (Fig. 1B). Cells from the outer ring of motility from the High cdG strain (Fig. 1B) exhibited the same phenotype as the parent High cdG strain upon reinoculation into a new plate (i.e., low motility) (see Fig. S3), arguing that the motility observed was not due to spontaneous suppressor mutants. An open question, then, was whether this strain exhibited flagellar motility under the conditions experienced in the light organ mucus. We therefore performed a competitive colonization assay between the High cdG strain and the amotile High cdG Δ*flrA* isogenic derivative. The High cdG strain outcompeted the amotile Δ*flrA* derivative, with a median advantage of ~10-fold (Fig. 4), supporting that the High cdG strain is indeed motile in the host.

10.1128/mbio.01671-22.3 FIG S3 High cdG motile cells are not suppressor mutants. A representative image of migration through soft (0.3%) agar for V. fischeri and indicated strains is shown. Download FIG S3, PDF file, 0.10 MB. Copyright © 2022 Isenberg et al. 2022 Isenberg et al. https://creativecommons.org/licenses/by/4.0/ This content is distributed under the terms of the Creative Commons Attribution 4.0 International license.

### Elevated c-di-GMP levels lead to altered aggregate morphology *in vivo*.

**FIG 5:**
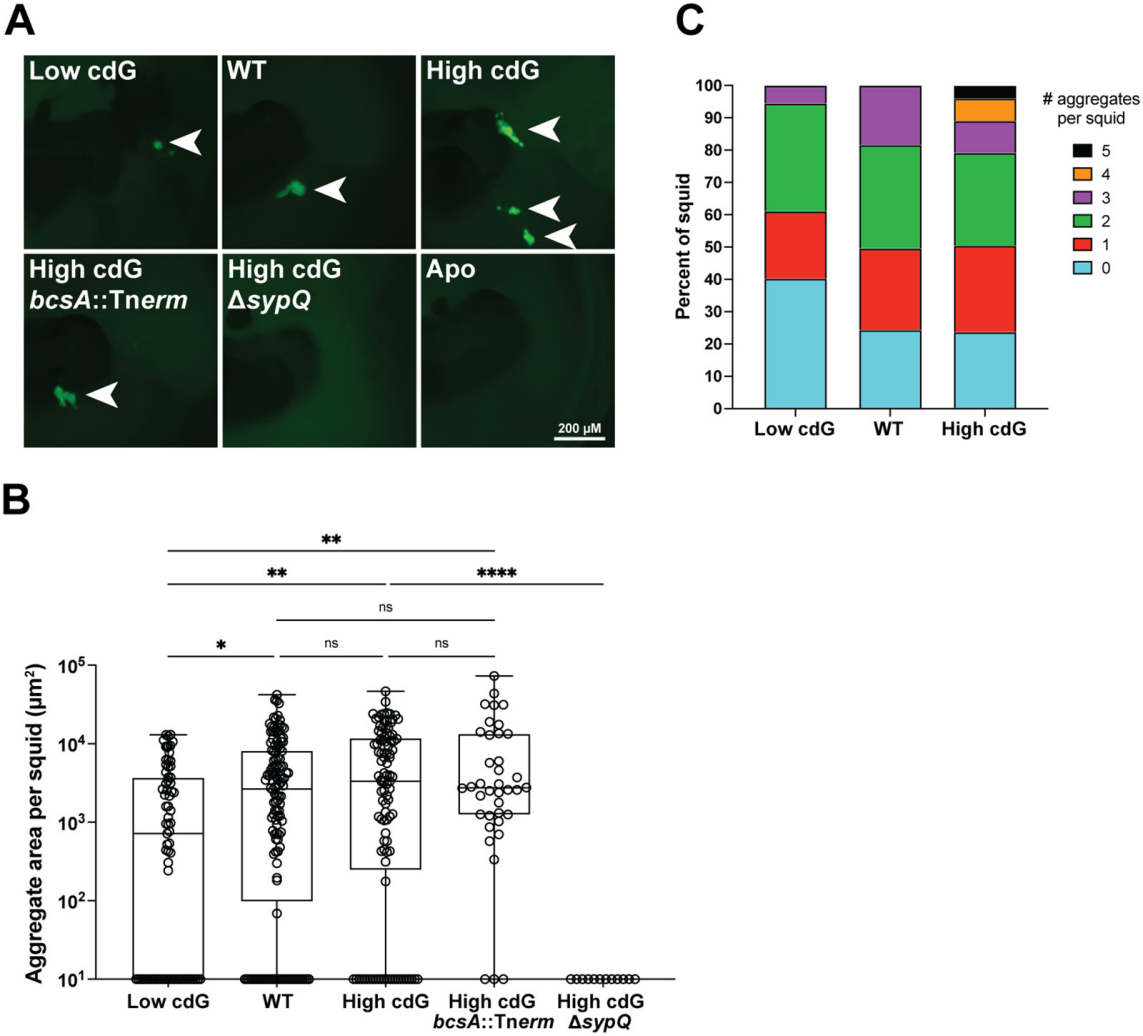
Elevated c-di-GMP levels lead to a greater number of V. fischeri aggregates in the squid host. (A) Representative fluorescence microscopy images of aggregates made by indicated V. fischeri strains carrying a constitutive GFP on the pVSV102 plasmid within the host mucus. Arrows indicate location of aggregates. (B) Quantification of aggregate area per squid for indicated V. fischeri strains carrying a constitutive GFP on the pVSV102 plasmid. Zero values are displayed at the bottom of the plot. Box-and-whisker plots represent the minimum, 25th percentile, median, 75th percentile, and maximum. Sample sizes from left to right are 73, 131, 101, 38, and 12 squid. A Kruskal-Wallis test was used for statistical analysis (ns, not significant; *, *P* < 0.04; **, *P* < 0.02; ****, *P* < 0.0001). (C) Quantification of number of aggregates formed per squid by indicated V. fischeri strains. Sample sizes from left to right are 73, 131, and 101 squid.

The V. fischeri-squid system provides an opportunity to combine genetic and imaging approaches to study the planktonic-to-biofilm transition during colonization of a symbiotic host. Symbiosis polysaccharide (Syp) is required for host colonization (5), while the distinct cellulose polysaccharide is not known to impact host phenotypes, so we questioned whether c-di-GMP would impact Syp-dependent aggregation *in vivo*. At 3 hpi, we examined *in vivo* biofilm aggregates using V. fischeri that constitutively expressed a fluorescent protein (green fluorescent protein [GFP] or mCherry). Compared to the Low cdG strain, the High cdG strain produced larger-sized aggregates, while the WT aggregates were an intermediate size (Fig. 5A and B). In >10% of the light organs examined, the High cdG strain produced four or more aggregates, whereas squid colonized by the WT strain had three aggregates at most (Fig. 5C).

Although the pFY4535 reporter plasmid was not designed for *in vivo* squid experiments, we attempted to use this tool to measure c-di-GMP levels in the host for the Low cdG, WT, or High cdG strain (28). AmCyan is produced constitutively from the plasmid, enabling us to locate the bacterial cells in the host. Turbo RFP as a reporter for c-di-GMP levels was substantially reduced in the Low cdG strain compared to WT, consistent with what we observed *in vitro* (Fig. 1; see also Fig. S4). Examination of the High cdG strain, however, revealed aggregate morphology that was not consistent with what we observed above with a constitutive GFP marking the cells in aggregates (Fig. 5). Specifically, we saw diffuse small groups of cells in the host in the High cdG/pFY4535 strain, rather than the aggregates that are produced by the High cdG/pVSV102 (GFP) strain. The High cdG/pFY4535 phenotype is the outlier, as the gene reporter assays described in the next paragraph use a constitutive mCherry to mark the cells, and their aggregate behavior matches that of the constitutive GFP in Fig. 5. Put together, our interpretation of these data is that the high level of reporter expression in the High cdG strain *in vivo* alters the aggregative behavior of the cells. While this effect prohibits accurate measurements of c-di-GMP levels in the host, it is consistent with a model in which high c-di-GMP levels in the strain are retained *in vivo*.

10.1128/mbio.01671-22.4 FIG S4 Multiple gene deletions alter *in vivo* c-di-GMP levels. (A) Quantification of c-di-GMP levels for indicated V. fischeri strains using the pFY4535 c-di-GMP reporter plasmid in aggregates within the host mucus. Samples sizes from left to right are 9 and 5 aggregates. A Mann-Whitney test was used for statistical analysis (***, *P* ≤ 0.01). (B) Representative fluorescent microscopy images of squid light organs containing indicated V. fischeri strains carrying the pFY4535 c-di-GMP reporter plasmid. Arrows indicate the location of aggregates. Download FIG S4, PDF file, 0.2 MB. Copyright © 2022 Isenberg et al. 2022 Isenberg et al. https://creativecommons.org/licenses/by/4.0/ This content is distributed under the terms of the Creative Commons Attribution 4.0 International license.

**FIG 6:**
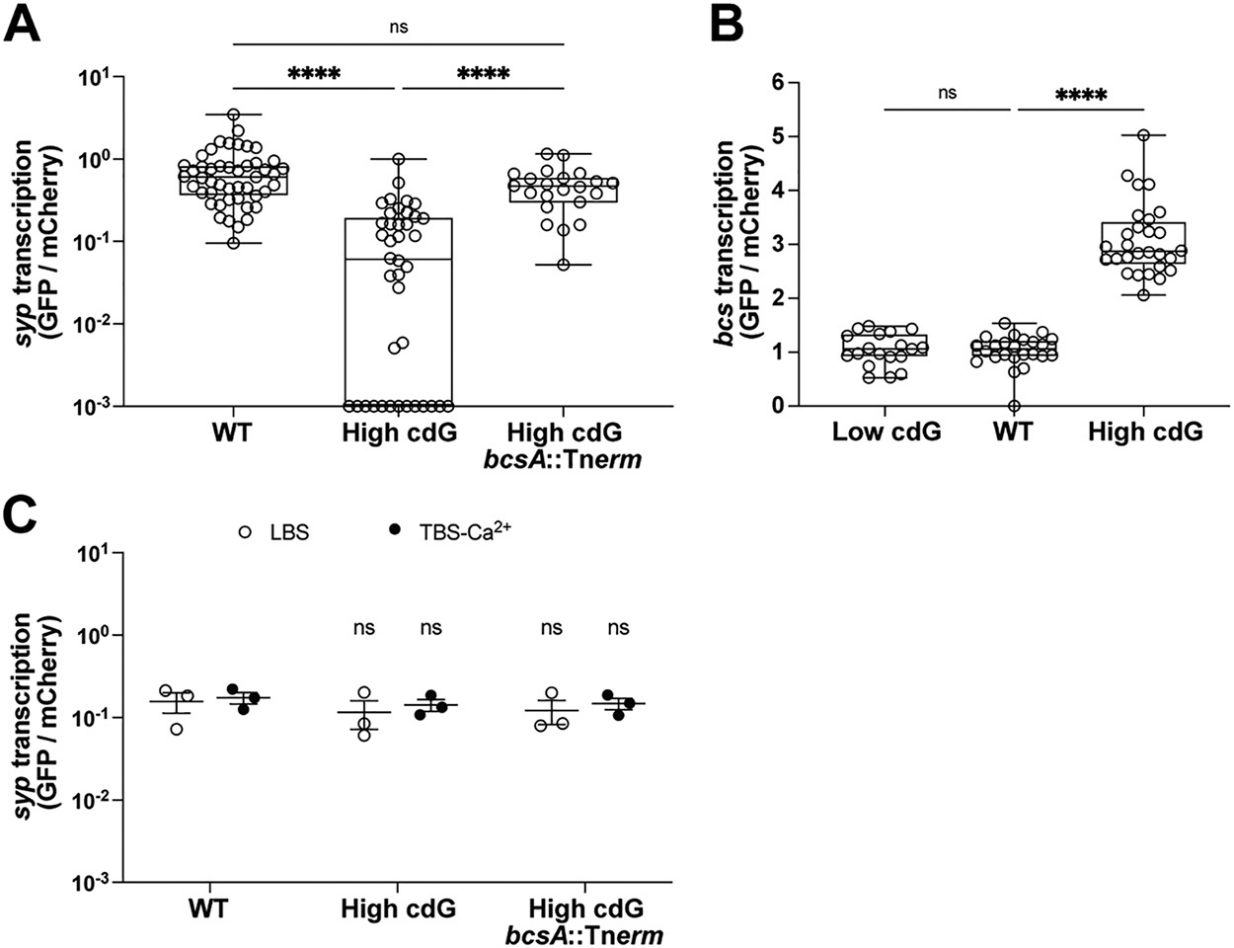
c-di-GMP downregulates *syp* via BcsA in host mucus but not in culture. (A) Quantification of *syp* transcription for indicated V. fischeri strains using the pM1422 *sypA*′-*gfp*^+^ transcriptional reporter plasmid in aggregates within the host mucus. Sample sizes from left to right are 47, 40, and 22 aggregates. (B) Quantification of *bcs* transcription for indicated V. fischeri strains using the pRYI063 *bcsQ*′-*gfp*^+^ transcriptional reporter plasmid in aggregates within the host mucus. Sample sizes from left to right are 19, 24, and 28 aggregates. For panels A and B, box-and-whisker plots represent the minimum, 25th percentile, median, 75th percentile, and maximum. A Kruskal-Wallis test was used for statistical analysis (ns, not significant; ****, *P* < 0.0001). (C) Quantification of *in vitro syp* transcription in LBS and TBS-Ca^2+^ for the indicated V. fischeri strains carrying the pM1422 *sypA*′*-gfp*^+^ transcriptional reporter plasmid. For each strain, *n *=* *3 biological replicates. Points represent each replicate, average bars represent the means of replicates, and error bars represent the standard errors of the mean. One-way ANOVA was used for statistical analysis (ns, not significant relative to the WT strain in the same media). For panels A to C, constitutive mCherry was used to normalize GFP to cell density.

To test whether the Syp polysaccharide was required for the pattern of multiple bacterial aggregates we observed in the High cdG strain, we deleted the gene encoding the structural Syp protein SypQ in the High cdG background. This strain failed to colonize the host robustly at 48 hpi (see Fig. S5B) and did not form detectable aggregates (Fig. 5A and B), demonstrating that Syp-dependent biofilm is required for *in vivo* aggregation in the High cdG background. We sought to determine whether transcription of the *syp* locus was altered in the High cdG strain. Activity of a *sypA′-gfp^+^* reporter was examined during the aggregation phase, where we found that in the host, *sypA′-gfp^+^* reporter activity was substantially reduced in the High cdG strain compared to the WT parent (Fig. 6A; see also Fig. S6A). High levels of c-di-GMP therefore negatively impact the expression of the symbiosis polysaccharide locus *in vivo*. Notably, when we sought to determine whether the High cdG strain had altered *sypA′-gfp^+^* reporter activity *in vitro*, we found no significant effects in LBS medium or in TBS-Ca^2+^ medium that leads to a higher basal *syp* level (Fig. 6C) (33). Therefore, c-di-GMP exerts a host-specific effect on symbiotic biofilm expression.

10.1128/mbio.01671-22.5 FIG S5 Absence of the cellulose synthase BcsA does not rescue the high c-di-GMP strain colonization defect. (A) Quantification of squid colonization levels at 18 hpi by indicated V. fischeri strains and aposymbiotic (Apo) control. Sample sizes from left to right are 14, 25, 17, and 13 squid. (B) Quantification of squid colonization levels at 48 hpi by indicated V. fischeri strains. Sample sizes from left to right are 68, 16, 75, 56, 17, and 65 squid. For panels A and B, data for WT, High cdG, and Apo groups are the same as in Fig. 3. Box-and-whisker plots represent the minimum, 25th percentile, median, 75th percentile, and maximum. A Kruskal-Wallis test was performed for statistical analysis for squid that were introduced to bacteria (ns, not significant; **, *P* = 0.002; ****, *P* < 0.0001). Download FIG S5, PDF file, 0.1 MB. Copyright © 2022 Isenberg et al. 2022 Isenberg et al. https://creativecommons.org/licenses/by/4.0/ This content is distributed under the terms of the Creative Commons Attribution 4.0 International license.

10.1128/mbio.01671-22.6 FIG S6 High c-di-GMP-dependent Bcs expression inhibits *syp* expression in bacterial aggregates within the host mucus. (A) Representative fluorescent microscopy images of squid light organs containing indicated V. fischeri strains carrying the pM1422 *sypA*′-*gfp*^+^ transcriptional reporter plasmid. (B) Representative fluorescent microscopy images of squid light organs containing indicated V. fischeri strains carrying the pRYI063 *bcsQ*′-*gfp*^+^ transcriptional reporter plasmid. Download FIG S6, PDF file, 0.4 MB. Copyright © 2022 Isenberg et al. 2022 Isenberg et al. https://creativecommons.org/licenses/by/4.0/ This content is distributed under the terms of the Creative Commons Attribution 4.0 International license.

### High levels of c-di-GMP repress *syp* polysaccharide transcription via the cellulose polysaccharide and in a host-dependent manner.

In previous studies in the squid host, larger bacterial aggregates have correlated with an upregulation of *syp* gene transcription and a higher propensity for colonization initiation and competitive fitness (5, 34, 35). In this case, however, the larger aggregates of the High cdG strain have reduced *sypA′-gfp^+^* reporter activity and exhibit poor colonization (Fig. 2 and Fig. 6A; see also Fig. S6A). We therefore sought to determine whether there was another component of the aggregate that may contribute to the morphology observed but that may not enhance colonization ability in the same way that additional Syp polysaccharide promotes colonization. A candidate for this component is cellulose, as we demonstrated above that cellulose is upregulated in the High cdG strain (Fig. 1C and D). Therefore, we sought to determine whether there may be an interaction between the cellulose and Syp polysaccharide biosynthetic pathways. We measured *bcsQ* transcription *in vivo* using a fluorescent transcriptional reporter (*bcsQ′-gfp^+^*), and we determined that *bcsQ′-gfp^+^* reporter activity was elevated in bacterial aggregates in the host mucus in the High cdG strain compared to WT (Fig. 6B; see also Fig. S6B). Using a *bcsA*::Tn*erm* transposon insertion mutant that interrupts cellulose synthase, we examined colonization in the context of the High cdG background. Interruption of *bcsA* did not improve colonization at 18 or 48 hpi compared to the High cdG strain (see Fig. S5). However, compared to the High cdG strain, a *bcsA* derivative exhibited higher *sypA′-gfp^+^* activity *in vivo* (Fig. 6B; see also Fig. S6B), yet no difference in *sypA′-gfp*^+^ activity was observed *in vitro* with the same strains, even with *syp* induced by added calcium chloride (Fig. 6C) (33). These data present a novel connection between transcriptional control of polysaccharide systems that occurs specifically in the host.

## DISCUSSION

### Cyclic di-GMP impacts multiple pathways during beneficial colonization.

Our study was motivated by the question of how c-di-GMP impacts a beneficial microbe-host association. To address this question, we focused on the V. fischeri-squid mutualism. This well-studied binary association allows us to examine individual stages of colonization using strains deleted for 6 to 7 genes and that consequently have altered levels of c-di-GMP. We found that a strain with elevated levels of c-di-GMP exhibited striking defects during host colonization. The High cdG strain formed aggregates that trended larger (Fig. 4), had altered host-specific polysaccharide gene expression in the aggregates (Fig. 6A and B; see also Fig. S6), exhibited reduced flagellar motility (Fig. 1B), and displayed a significant colonization defect as enumerated by bacterial counts in the host as early as 18 hpi (Fig. 2). Bacterial counts did not recover by 48 hpi, suggesting that high levels of c-di-GMP confer both an initiation defect and an accommodation defect on the colonizing strains (36), as discussed further below. Colonization of the High cdG strain was rescued by overexpression of a heterologous PDE that does not have significant homology to proteins in V. fischeri (Fig. 3D), strongly supporting that the observed effects are due to the c-di-GMP levels and not to individual functions of the gene products affected in the multiple deletion strains.

Our results parallel work in several pathogenic bacteria, where elevated c-di-GMP levels are correlated with a decrease in virulence (3, 37–40). In Yersinia pestis, the small number of relevant enzymes made it possible to remove all of the DGCs or PDEs. Deletion of both genes for the functional DGCs effectively eliminated c-di-GMP in the cell (measured by liquid chromatography-mass spectrometry), yet virulence in mouse infection models was not affected (40). However, deletion of the single functional PDE gene, *hmsP*, led to ~10-fold higher c-di-GMP levels and led to reduced virulence in a subcutaneous infection model (40). In V. cholerae, elevated c-di-GMP (visualized by two-dimensional thin-layer chromatography) led to 5-fold reduced competitive colonization in the infant mouse small intestine model (39). In that study, the authors tested whether the reduction was due to overexpression of biofilm (VPS), but it was not, and they attributed the phenotype to the resulting virulence gene expression downstream of c-di-GMP. We note that the magnitude of the effect we observe in V. fischeri colonization of bobtail squid is substantially higher: >100-fold in a single-strain colonization assay. It therefore seems likely that this aspect of mutualism is broadly shared with pathogenic colonization, and we expect there to be strong constraints to retain c-di-GMP levels low during the initiation of the host colonization process. There are also likely to be beneficial symbiosis-specific adaptations within c-di-GMP signaling. For example, the zebrafish gut symbiont Aeromonas veronii responds to host amino acids to inhibit a diguanylate cyclase, thereby stimulating bacterial motility into the host (41). It remains to be seen whether there are similar specific adaptations in the V. fischeri-squid symbiosis.

We were surprised to find that substantially lowering levels of c-di-GMP (~9-fold) (Fig. 1A) led to no significant defect in host colonization. In the Low cdG strain, we detected slightly smaller bacterial aggregates in the host mucus (Fig. 5), but no consequent diminution of bacterial counts in the light organ at 18 hpi or 48 hpi (Fig. 2). The Low cdG strain similarly exhibited no competitive colonization defect (see Fig. S1). When examining transcriptional reporters for the symbiosis polysaccharide gene *sypA* or the cellulose biosynthesis gene *bcsQ in vivo*, the Low cdG strain resembled the wild-type parent (Fig. 6A and B; see also Fig. S6). We note that colonization was intact despite this strain exhibiting strong biofilm and motility phenotypes *in vitro* (Fig. 1), thus emphasizing the importance of studying these phenotypes in the relevant host system. The key bacterial behaviors during the initial stages in the host include adhering to host mucus and then cilia within the mucus (10); forming the Syp-dependent aggregates (5, 9–11); migrating toward the host pores in a manner independent of flagellar motility (4, 12); employing flagellar motility and chemotaxis to swim toward chitin oligosaccharides released by the host (8, 14–17); and resisting host innate immune attacks as they traverse the pore into the duct, antechamber, bottleneck, and then crypts of the light organ (42–45). These processes take 18 to 24 h and encompass the “initiation” and “accommodation” phases of the symbiosis. Around 18 to 24 h, the interaction shifts to the “persistence” phase, where quorum sensing stimulates bioluminescence in the host, and a daily (diel) rhythm ensues in which bacteria consume nutrient sources provisioned from the host squid. We observe specific initiation defects in biofilm reporter gene expression, biofilm aggregate number and architecture, flagellar motility, and bacterial counts in the host at 18 hpi. We additionally observe an accommodation defect in that bacterial counts do not recover by 48 hpi, arguing that the High cdG strain continues to inhibit expansion in the crypts following the initial defect. The accommodation phenotype is unlikely to be due to flagellar motility, as most bacteria lose their flagella during the mature association (8). Therefore, these effects may be due to regulation of biofilm genes in the host (7) or may be due to other unrecognized effects in the High cdG strain.

By using strains with altered c-di-GMP levels, we have uncovered regulatory differences in transcriptional reporters in culture versus the host. In culture, we observed a 3.2-fold increase in *bcsQ′-gfp^+^* reporter activity in the WT strain relative to the Low cdG strain (Fig. 1D). However, in the animal, we observed similar *bcsQ′-gfp^+^* reporter activity in the two strains (Fig. 6B), suggesting that the lower *bcsQ′-gfp^+^* activity of the WT strain may occur in response to the host environment. Similarly, the upregulation of *bcs* gene expression in the background of elevated c-di-GMP conditions led to a substantial downregulation of *sypA′-gfp*^+^ reporter activity in the host, yet no comparable effect was observed in two distinct culture conditions (Fig. 6). Given the multiple cases in which gene expression in the host is distinct from that observed in culture, it remains an intriguing question as to how the host influences pathways linked to c-di-GMP signaling.

If the Low cdG strain thrives in the host under conditions where levels of c-di-GMP remain low, is c-di-GMP required for V. fischeri biology in the squid? Given that there are another twenty-one DGCs and five putative bifunctional DGC/PDEs still present in the Low cdG strain, our results do not preclude a requirement for c-di-GMP during colonization. It remains possible that additional DGCs perform localized functions that were not addressed in the present study and/or that DGCs play critical roles at later stages in the colonization process.

### Construction of multiple-gene deletions in *V. fischeri* is an effective tool to tackle gene families.

The recent development of a method for construction of serial deletions (26) enabled the construction of multiple-mutant strains such as the Low cdG and High cdG strains in this study. We have further extended the method to allow for detection of barcoded deletions in competitive colonization experiments (46). The work in this study highlights the utility of such approaches to interrogate gene families. There are many instances in which deletions of multiple genes could clarify the role of various biological processes. Previously, we identified a role for chemotaxis toward chitin oligosaccharides as a key developmental stage during squid colonization (16). Efforts from our group and others have studied 23 V. fischeri chemoreceptors through a combination of single and multiple mutants (47–49), but there are a total of 43 methyl-accepting chemoreceptors encoded in the ES114 genome, and the receptor(s) for chitin oligosaccharides remain elusive. Generation of multiple mutant strains has been especially valuable to dissect gene families in V. cholerae (50), and our work suggests that a similar approach will be useful in V. fischeri.

### Elevated c-di-GMP levels diminish, but do not eliminate, flagellar motility *in vivo*.

The High cdG strain has a significant soft agar motility defect (Fig. 1B), and amotile strains are at a significant colonization disadvantage compared to WT. Our competition of the High cdG strain with the isogenic Δ*flrA* derivative revealed a 15-fold advantage for the former during squid colonization (Fig. 4). Our interpretation of this result is that the High cdG strain retains the ability to be motile in the host despite the elevated c-di-GMP levels. The magnitude of the competitive advantage (15-fold) is diminished compared to that of the wild-type strain competing against a Δ*flrA* strain (>1,000-fold) (46, 51). Therefore, it seems likely that c-di-GMP does impact motility in the host, but not to the extent that it fully prohibits colonization.

### Cyclic di-GMP has a qualitative effect on the nature of the exopolysaccharide in animal tissue.

The increased number of aggregates observed in the High cdG strain in squid is, to our knowledge, the first time such a phenotype has been observed (Fig. 5). Hypermotile mutants were noted to exhibit more diffuse aggregates on the light organ surface, though these were often smaller than their WT counterparts (15). Multiple studies have noted larger *in vivo* aggregates upon overexpression of biofilm activator RscS or removal of biofilm inhibitor BinK (5, 35). In both cases, it was shown that Syp was the relevant pathway induced, and the cells within the resulting larger aggregates were functional to outcompete those from WT aggregates. The morphology and gene expression in High cdG aggregates are significantly more complicated and implicate an interaction between cellulose and Syp exopolysaccharides. We showed that cellulose on its own is not required for colonization (see Fig. S5B). However, the high c-di-GMP condition revealed a role for the *bcs* (cellulose synthase) locus in regulating *syp* (mirroring results obtained in specific agar conditions (see reference 52) (Fig. 6A; see also Fig. S6A)). Therefore, while cellulose production on its own does not impact colonization, in the High cdG background it significantly impacts expression of *syp*. In Pseudomonas aeruginosa, roles for multiple exopolysaccharide systems in biofilm formation have been explored, for example as the Psl and Pel systems have distinct but also overlapping roles in attachment and biofilm formation (53). In V. fischeri, our work supports data in the literature that Syp is the key system for *in vivo* biofilm formation and that the cellulose system is dispensable for host colonization. At the same time, this study reveals a novel transcriptional interaction between two exopolysaccharide systems. This interplay may impact the formation of multiple-exopolysaccharide biofilms during later stages of host colonization or under environmental conditions that have yet to be explored, and points to a novel regulatory mechanism that connects biofilm regulation in an animal host.

In conclusion, this work identified a role for c-di-GMP regulation during the initiation of a mutualism, identified specific developmental stages in the host that were regulated by altered c-di-GMP levels, and revealed a transcriptional interplay between multiple exopolysaccharide systems that is apparent only in the host. Our study therefore lays the groundwork for future investigations into how this widespread bacterial compound influences bacterial behaviors beyond pathogenesis to impact microbiome behavior and interactions with symbiotic hosts.

## MATERIALS AND METHODS

### Bacterial strains, plasmids, and media.

V. fischeri and E. coli strains used in this study are listed in Table 1. Plasmids used in this study are listed in Table 2. V. fischeri strains were grown at 25°C in Luria-Bertani salt (LBS) medium (per L: 25 g of Difco LB broth [BD], 10 g of NaCl, 50 mL of 1 M Tris buffer [pH 7.5]) or TBS (per L: 10 g of tryptone [VWR], 20 g of NaCl, 35 mM MgSO_4_ [where noted], 10 mM calcium chloride [where noted], 50 mM 1 M Tris buffer [pH 7.5]) where noted. Escherichia coli strains used for cloning and conjugation were grown at 37°C in Luria-Bertani (LB) medium (per L: 25 g of Difco LB broth [BD]). When needed, antibiotics were added to the media at the following concentrations: kanamycin, 100 μg/mL for V. fischeri and 50 μg/mL for E. coli; chloramphenicol, 1 or 5 μg/mL for V. fischeri and 25 μg/mL for E. coli; erythromycin, 2.5 or 5 μg/mL for V. fischeri; gentamicin, 2.5 μg/mL for V. fischeri and 5 μg/mL for E. coli; trimethoprim, 10 μg/mL for V. fischeri; and spectinomycin, 160 μg/mL in LB for V. fischeri. When needed, thymidine was added at 0.3 mM for E. coli. Growth medium was solidified using 1.5% agar when needed. For Congo red agar, 40 μg/mL Congo red and 15 μg/mL Coomassie blue were added to LBS. For X-Gal (5-bromo-4-chloro-3-indolyl-β-d-galactopyranoside) agar, 20 μg/mL X-Gal was added to LBS. Plasmids were introduced from E. coli strains into V. fischeri strains using standard techniques (54, 55).

**TABLE 1:** Strains used in this study

Strain	Genotype	Source or reference(s)
V. fischeri		
KV4674 = ES114	Natural isolate, squid light-organ (Visick Lab Stock)	57, 58
KV8069	KV4674/Δ*sypQ*::FRT-Cam	33
KV8408	KV4674/*bcsA*::Tn*erm*	26
KV8920	KV4674/Δ*VF_1639*::FRT-Spec	59
KV8932	KV4674/Δ*VF_1200*::FRT-Cam	60
KV8969	KV4674/Δ*pdeV*::FRT	23
KV9599 = Δ7DGC (Low cdG)	Δ*VF_1200*::FRT Δ*mifA*::FRT Δ*VF_1245*::FRT Δ(*VF_A0342-VF_A0343*)::FRT Δ*VF_1639*::FRT Δ*mifB*::FRT-Spec	This study
KV9601 = Δ6PDE (High cdG)	Δ*pdeV*::FRT Δ*VF_2480*::FRT Δ*binA*::FRT Δ*VF_0087*::FRT Δ*VF_1603*::FRT Δ*VF_A0506*::FRT-Trim	This study
KV9767	KV9601 *bcsA*::Tn*erm*	This study
KV9769	KV9601 Δ*sypQ*::FRT-Cam	This study
MJM1100 = ES114	Natural isolate, squid light-organ (Mandel Lab Stock)	57, 58
MJM1438	MJM1100/pM1422	35
MJM4009	MJM1100/pFY4535	This study
MJM4135	KV9599/pFY4535	This study
MJM4137	KV9601/pFY4535	This study
MJM4138	KV9560/pFY4535	This study
MJM4139	KV9569/pFY4535	This study
MJM4140	KV9599/pVSV102	This study
MJM4142	KV9601/pVSV102	This study
MJM4198	KV9599/pM1422	This study
MJM4200	KV9601/pM1422	This study
MJM4308	KV9767/pFY4535	This study
MJM4310	KV9769/pFY4535	This study
MJM4312	KV9767/pVSV102	This study
MJM4314	KV9769/pVSV102	This study
MJM4322	KV9767/pM1422	This study
MJM4484	MJM1100 *bcsA*::Tn*erm*	This study
MJM4592	KV9601/pVSV103	This study
MJM4593	MJM1100/pRYI063	This study
MJM4594	KV9599/pRYI063	This study
MJM4595	KV9601/pRYI063	This study
MJM4596	KV9767/pRYI063	This study
MJM4597	MJM4484/pRYI064	This study
MJM4603	KV9601 Δ*flrA*::*erm*-bar	This study
MJM4630	MJM1100/pRYI064	This study
MJM4631	KV9601/pRYI064	This study
MJM4632	MJM1100/pFY4535; pRYI064	This study
MJM4634	KV9601/pVSV105	This study
MJM4635	MJM1100/pFY4535; pVSV105	This study
MJM4636	KV9601/pFY4535; pVSV105	This study
E. coli		
KV6937	DH5α/pLostfox-Kan	25
KV8052	π3813/pKV496	26
MJM542	DH5α λpir/pVSV102	47
MJM552	DH5α λpir/pVSV103	47
MJM579	DH5α λpir/pVSV105	47
MJM1422	DH5α λpir/pM1422	35
CAB1516	DH5α λpir/pVSV105	47
MJM3999	NEB5α/pFY4535	28
MJM4580	DH5α λpir/pRYI063	This study
MJM4625	DH5α λpir/pRYI064	This study

**TABLE 2:** Plasmids used in this study

Plasmid	Description	Source or reference
pEVS104	Conjugal helper plasmid (Kan^r^)	54
pFY4535	c-di-GMP reporter plasmid (Gent^r^)	28
pJJC4	Vector carrying *tfoX* and *litR* genes (Cam^r^)	61
pKV494	Vector carrying FRT-Erm^r^	26
pKV495	Vector carrying FRT-Cam^r^	26
pKV496	pLostfoX-Kan backbone containing the FLP recombinase (Kan^r^)	26
pKV521	Vector carrying FRT-Spec^r^	26
pLostfoX-Kan	TfoX induction vector for chitin-pathway transformation (Kan^r^)	25
pM1422	pTM267 *sypA*′-*gfp*^+^ (Cam^r^)	35
pMCL2	Vector carrying FRT-Trim^r^	26
pRYI063	pTM267 *bcsQ*′-*gfp*^+^ (Cam^r^)	This study
pRYI064	pVSV105 carrying *VC1086* (Cam^r^)	This study
pVSV102	Constitutive GFP (Kan^r^)	62
pVSV103	Constitutive LacZ (Kan^r^)	62
pVSV105	Complementation vector (Cam^r^)	47

### DNA synthesis and sequencing.

Primers used in this study are listed in Table 3 and were synthesized by Integrated DNA Technologies (Coralville, IA). Full inserts for cloned constructs and gene deletions were confirmed by Sanger Sequencing at Functional Biosciences via UW-Madison. Sequence data were analyzed using Benchling. PCR to amplify constructs for cloning and sequencing were performed using Q5 High-Fidelity DNA polymerase (NEB), OneTaq DNA polymerase (NEB), or MilliporeSigma Novagen KOD DNA polymerase. Diagnostic PCR was performed using GoTaq polymerase (Promega) or OneTaq DNA polymerase.

**TABLE 3:** Primers used in this study

Primer	Sequence (5′–3′)^*a*^	Notes
2089	CCATACTTAGTGCGGCCGCCTA	Forward primer to amplify antibiotic resistance cassette
2090	CCATGGCCTTCTAGGCCTATCC	Reverse cassette to amplify antibiotic resistance cassettes
2537	AGTGCCTCATATAAGGGTTAAG	Outside forward PCR primer for the Δ(*VF_A0342-A0343*) construct
2538	taggcggccgcactaagtatggTGTTACGTCAACAACCTTTTGG	Inside reverse PCR primer for the Δ(*VF_A0342-A0343*) construct
2543	ggataggcctagaaggccatggATGGCGGATACTGCATTGTATC	Inside forward PCR primer for the Δ(*VF_A0342-A0343*) construct
2544	CCAAATCGACAGAGATCCCC	Outside reverse PCR primer for the Δ(*VF_A0342-A0343*) construct
2593	GCCAATGCAGATATGACAAAGG	Outside forward PCR primer for the Δ*VF_1245* construct
2558	taggcggccgcactaagtatggTAAGCGCTGAAGACGAGATTG	Inside reverse PCR primer for the Δ*VF_1245* construct
2559	ggataggcctagaaggccatggATTGAACAAGCAGATAAGGCGC	Inside forward PCR primer for the Δ*VF_1245* construct
2560	GCATTCATTAGTGATGTGCGTG	Outside reverse PCR primer for the Δ*VF_1245* construct
2905	ggataggcctagaaggccatggCACTTCGTGTTAAAGAATTTATAC	Confirmation of Δ*VF_1639*
2564	CTAACCATTCATGCAAGAACC	Confirmation of Δ*VF_1639*
2577	TAAAGCAGCAGCTGAGCAAG	Confirmation of Δ*VF_1200*
2580	GTTTGGTGAGTACCAATCGC	Confirmation of Δ*VF_1200*
2585	CAAAGCATCAAAAACTCACTTGC	Outside forward PCR primer for the Δ*VF_0087* construct
2586	taggcggccgcactaagtatggAATGCTTTGAACACTAAATGAGAG	Inside reverse PCR primer for the Δ*VF_0087* construct
2587	ggataggcctagaaggccatggGGTGAGCCTAAATCGTTACAC	Inside forward PCR primer for the Δ*VF_0087* construct
2588	AACGAACGCCACAAACTCAC	Outside reverse PCR primer for the Δ*VF_0087* construct
944	CGCATACGTTTCTACCGTTTC	Confirmation of Δ*VF_0087*
945	CCAAGCTGAACGAAGTGGAC	Confirmation of Δ*VF_0087*
2598	CACTGATGGTTTAGAGCTTGG	Outside forward PCR primer for the Δ*mifA* construct
2599	taggcggccgcactaagtatggCAGCTCTGTTTGATATAGTGTCC	Inside reverse PCR primer for the Δ*mifA* construct
2600	ggataggcctagaaggccatggAGTGAAATGGATCAAATCGCATG	Inside forward PCR primer for the Δ*mifA* construct
2601	TTGATGCGAGATTGAAATACGC	Outside reverse PCR primer for the Δ*mifA* construct
2618	AGAGCAGCTCGTGAGTTATC	Confirmation of Δ*pdeV*
2621	CTTCCATAGTAAGAACCTCTGC	Confirmation of Δ*pdeV*
2648	GCATGTGTACCAAGTAAGCTTG	Outside forward PCR primer for the Δ*VF_A0506* construct
2649	taggcggccgcactaagtatggCATTGTCCATTCATAAGATGGGC	Inside reverse PCR primer for the Δ*VF_A0506* construct
2650	ggataggcctagaaggccatggCGAGGTCAACAATTTGACCC	Inside forward PCR primer for the Δ*VF_A0506* construct
2651	GGCATTGATGAGTCTTAAGCTG	Outside reverse PCR primer for the Δ*VF_A0506* construct
2674	CTAAAGTGCACAAACCATGGC	Outside forward PCR primer for the Δ*VF_1603* construct
2675	taggcggccgcactaagtatggGTCTAAGATTGGTTGACGAGC	Inside reverse PCR primer for the Δ*VF_1603* construct
2676	ggataggcctagaaggccatggTCTGCCAAATGGGTAGATGAG	Inside forward PCR primer for the Δ*VF_1603* construct
2677	TTATTGCAGGTGCTGCACTG	Outside reverse PCR primer for the Δ*VF_1603* construct
2678	TTGGCCACGGTGATCGAAAG	Outside forward PCR primer for the Δ*VF_2480* construct
2679	taggcggccgcactaagtatggTATCGGTTGTCTGGCAACG	Inside reverse PCR primer for the Δ*VF_2480* construct
2680	ggataggcctagaaggccatggACCTACCAAGAAGCGGTTAAG	Inside forward PCR primer for the Δ*VF_2480* construct
2681	TTGCTGCTAATCGTGACAGC	Outside reverse PCR primer for the Δ*VF_2480* construct
1152	CAGTAGAGTCACTACCGTTG	Outside forward PCR primer for the Δ*binA* construct
2714	taggcggccgcactaagtatggTTGTTCAGGAATGTCGATGGC	Inside reverse PCR primer for the Δ*binA* construct
2715	ggataggcctagaaggccatggCACTTTGTGTAATGGGCACTC	Inside forward PCR primer for the Δ*binA* construct, inside forward
2716	GCCTCTGGATTTGGATAGTG	Outside reverse PCR primer for the Δ*binA* construct
850	gggcccAGAATAAACTGCTACTATCT	Confirmation of Δ*binA*
445	AAAACAGTAATCAGGGTGGAACG	Confirmation of Δ*binA*
2753	ATAGATGGTAAAGCGTCATACC	Outside forward PCR primer for the Δ*mifB* construct
2754	taggcggccgcactaagtatggACGTAAGCTCACATATGGGC	Inside reverse PCR primer for the Δ*mifB* construct
2755	ggataggcctagaaggccatggTTTACCGTTGCTGATGAACATATG	Inside forward PCR primer for the Δ*mifB* construct
2756	CAAACCATTGAACGCCATGATC	Outside reverse PCR primer for the Δ*mifB* construct
M13 For. (−20)	GTAAAACGACGGCCAGT	For amplification of gene inserts in pVSV105
M13 Rev. (−48)	AGCGGATAACAATTTCACACAGG	For amplification of gene inserts in pVSV105
RYI526	GTGCCCATTAACATCACCATCTAATTCA	Primer to confirm replacement of Kan^R^ cassette with promoter of choice
RYI560	GATATCAATGAGGTAATGCTTTTATTTTTTGCGAA	Forward primer to confirm transposon insertion in *bcsA*
RYI561	GCATAATTCTTACTTGGTTGCATGGTTTG	Reverse primer to confirm transposon insertion in *bcsA*
RYI576	gctaCCCGGGggtttgtggcatgacgatcact	Forward primer to amplify P*bcsQ* with XmaI restriction site
RYI577	cacgTCTAGAactaactttattctccaatgtattaactacacattttgg	Reverse primer to amplify P*bcsQ* with XbaI restriction site
RYI578	CAGGTCGACTCTAGAGGATCCCC	Forward primer to linearize pVSV105 for Gibson assembly
RYI579	CAGGCATGCAAGCTTGAGTATTCTAT	Reverse primer to linearize pVSV105 for Gibson assembly
RYI580	tactcaagcttgcatgcctgGAAGAACACCATGGCAAAATAAAGCTC	Forward primer to amplify *VC1086* with 101 bp upstream for Gibson assembly with pVSV105
RYI581	gatcctctagagtcgacctgCGGTTAAGCGCTTTCAAAATATCGTTTTC	Reverse primer to amplify *VC1086* with 99 bp downstream for Gibson assembly with pVSV105
RYI584	GGTGATAGAGATTAATCGCCGCTATCC	Sequencing primer for *VC1086*
RYI585	GATAGCTTTCAGAACCCAGCACCT	Sequencing primer for *VC1086*
RYI586	GTTACTGAGCGTCGCGGAGG	Sequencing primer for *VC1086*
RYI587	TTATGGAGATAGTTTGTTATGGAGATAGTTCAGGA	Sequencing primer for *VC1086*

a 5′-end lowercase sequences are not complementary to the amplified templates. For RYI576 and RYI577, uppercase indicates the restriction endonuclease recognition sites.

### Construction of multiple gene deletion mutants.

The multiple DGC and PDE mutants were constructed as follows. First, single gene deletions were generated using previously described PCR-SOE and *tfoX*-induced transformation methods (26, 56); some of these mutants have been previously described (23, 52). Briefly, regions flanking a gene of interest were amplified using primers listed in Table 3 and joined to an antibiotic-resistance cassette (e.g., Trim^r^/Erm^r^/Spec^r^) flanked by Flp-recombinase target (FRT) sites that was amplified with primers 2089 and 2090. The resulting fused PCR product was transformed into a V. fischeri strain (generally ES114) that contained a TfoX-overproducing plasmid (pLostfoX or pLostfoX-Kan plasmid (25, 56). Selection for the integrants was conducted on selective agar. The deletion/insertion was verified by PCR, generally with the outside primers used to generate the flanking fragments. Next, strains with multiple mutations were generated using genomic DNA from specific single mutants as follows. A *tfoX*-overexpression plasmid was introduced into a mutant of interest and transformed with genomic DNA containing a specific mutation, followed by selection for and verification of the mutation as described above. The resulting strain carrying a *tfoX*-overexpression plasmid could then serve as a recipient for the introduction of the next mutation. Because some mutants were generated using the same antibiotic resistance cassette, the Flp-encoding plasmid pKV496 was introduced as needed to induce the loss of the antibiotic resistance cassette and the retention of a 112-bp FRT scar in the gene. Following strain construction, the integrity of each deletion junction was verified with diagnostic primers listed in Table 3 to ensure that only recombination at local FRT sites occurred. As noted in the strain list (Table 1), the final cassette often was left in place. As an example, for Δ7PDE strain KV9601, the starting strain, KV8969 (23) (Δ*pdeV*::FRT) carrying *tfoX* plasmid pLostfoX-Kan was sequentially transformed with genomic DNA carrying the Δ*VF_2480*::FRT-Erm, Δ*binA*::FRT-Trim, and Δ*VF_0087*::FRT-Spec mutations. The cassettes were removed, and the *tfoX* plasmid reintroduced. Next, the Δ*VF_1603*::FRT-Trim mutation was introduced, and the cassette removed. Finally, the Δ*VF_A0506*::FRT-Trim mutation was introduced. Similar methods were used to generate KV9599 (Δ7DGC) and the derivatives of KV9601 that carry mutations in *sypQ* or *bcsA* (the latter of which was derived from strain KV8408, which carries a Tn mutation in *bcsA* [26]) rather than a cassette as described above).

### Construction of *bcsA*::Tn*erm* strain.

Genomic DNA isolated from KV9767 (Δ6PDE *bcsA*::Tn*erm*) was introduced into MJM1100 via transformation using pLostfoX-Kan (25, 56). Mutant candidates were selected using erythromycin and screened by PCR using RYI560 and RYI561 primers.

### Construction of the *bcsQ′*-*gfp*^+^ transcriptional reporter plasmid pRYI063.

The *bcsQ* promoter (*bcsQ* −444 to −29; P*bcsQ*) was amplified from MJM1100 genomic DNA using RYI576 and RYI577 primers that added flanking XmaI and XbaI restriction sites. The amplified P*bcsQ* and pTM267 were digested with XmaI and XbaI. Digested pTM267 was treated with Antarctic phosphatase. Digested P*bcsQ* was ligated into digested pTM267 using T4 DNA ligase (NEB). The ligation reaction was transformed into chemically competent DH5α λpir. Transformant candidates were selected using chloramphenicol and confirmed by Sanger sequencing using the RYI526 primer.

### Construction of the *VC1086* expression plasmid pRYI064.

pVSV105 was amplified using RYI578 and RYI579 primers. *VC1086*, along with 101 bp upstream and 99 bp downstream, was amplified from V. cholerae C6706 str2 genomic DNA using RYI580 and RYI581 primers. *VC1086* was assembled with pVSV105 by Gibson assembly using NEBuilder HiFi DNA Assembly Master Mix (NEB). The Gibson reaction was transformed into chemically competent DH5α λpir and transformant candidates were selected using chloramphenicol. Plasmid was screened by PCR using M13 For. (–20) and M13 Rev. (–48) primers. Plasmid was confirmed by Sanger sequencing using M13 For. (–20), M13 Rev. (–48), RYI584, RYI587, RYI585, and RYI586 primers.

### c-di-GMP quantification.

Strains were streaked on LBS agar and incubated overnight at 25°C. Liquid LBS was inoculated from single colonies and grown at 25°C overnight on a rotator. Next, 8 μL of liquid culture was spotted onto LBS agar, followed by incubation at 25°C for 24 h. Spots were resuspended in 1 mL of 70% Instant Ocean (IO). The optical density at 600 nm (OD_600_), TurboRFP (555-nm excitation/585-nm emission), and AmCyan (453-nm excitation/486-nm emission) for each resuspended spot were measured in triplicate using BioTek Synergy Neo2 plate reader. To calculate c-di-GMP levels, TurboRFP values (reports c-di-GMP levels) were normalized to AmCyan values (constitutively expressed). To compare c-di-GMP levels of strains in LBS versus IO, 1 mL of overnight LBS liquid cultures were pelleted at 8,000 × *g* for 10 min, resuspended in 600 μL of filter-sterilized IO (FSIO), and both the FSIO and the LBS samples were incubated at room temperature for 3 h. LBS and FSIO strains were pelleted at 8,000 × *g* for 10 min and resuspended in 600 μL of FSIO. The OD_600_, TurboRFP, and AmCyan for each sample were measured in triplicate as described above.

### Motility assay.

Strains were streaked on TBS and TBS-Mg^2+^ agar and incubated overnight at 25°C. Single colonies were inoculated into TBS and TBS-Mg^2+^ soft agar (0.3% agar), respectively, and incubated at 25°C for 5 h. Images of plates were taken using a Nikon D810 digital camera, and the diameter of migration was measured using ImageJ software.

### Isolation of cells from the outer edge of motility ring.

Strain was streaked on TBS-Mg^2+^ agar and incubated overnight at 25°C. A single colony was inoculated into TBS-Mg^2+^ soft agar (0.3% agar) and incubated at 25°C for 18 h. Cells from the outer edge of the resulting motility ring were streak purified on LBS. Isolates were then reinoculated into new TBS-Mg^2+^ motility agar as described above.

### Congo red assay.

Strains were streaked on LBS agar and incubated overnight at 25°C. Liquid LBS was inoculated with single colonies and grown at 25°C overnight on a shaker. Then, 4-μL spots of liquid culture were spotted on LBS Congo red agar, followed by incubation 24 h at 25°C. The spots were transferred onto white printer paper (26), and images were scanned as TIFF files. Congo red binding was quantified using ImageJ software.

### *syp* and *bcs* transcriptional reporter analysis *in vitro*.

Strains were streaked on LBS agar and incubated overnight at 25°C. Liquid medium was inoculated with single colonies and grown at 25°C for 24 h on a shaker. Next, 4-μL portions of overnight cultures were spotted onto TBS or TBS-Ca^2+^ agar plates and grown at 25°C for 24 h (52). The spots were imaged using a Zeiss Axio Zoom.v16 large-field 561 fluorescent stereo microscope, and fluorescence levels were measured using the Zen Blue Software polygon tool. To calculate gene promoter activity, GFP values (reports promoter activity) were normalized to mCherry values (constitutively expressed).

### Growth curves.

Strains were streaked on LBS agar and incubated overnight at 25°C. Liquid LBS was inoculated with single colonies of each strain in triplicate and grown at 25°C overnight on a rotator. Cultures were diluted 1:1,000 in LBS and grown at 25°C on a rotator to an OD_600_ of ~0.5. Cultures were diluted 1:100 in LBS (*n* = 6 technical replicates per culture) in a 96-well microtiter plate. The OD_600_ was measured every 15 min for 20 h at 25°C using a BioTek Synergy Neo2 plate reader. The doubling time was calculated in Prism using a nonlinear regression analysis of each strain during exponential phase.

### Squid single strain colonization assay.

*E. scolopes* hatchlings were colonized with approximately 10^3^ to 10^5^ CFU/mL of bacteria according to a standard procedure (29). At 18 and 48 hpi, the luminescence of the hatchlings was measured by using a Promega GloMax 20/20 luminometer, and the hatchlings were euthanized by storage at −80°C. CFU counts per light organ were determined by plating homogenized euthanized hatchlings and counting colonies.

### Squid competitive colonization assay.

Strains were grown overnight in liquid LBS at 25°C on a rotator. Strains were diluted 1:80 in liquid LBS and grown to an OD_600_ of ~0.2. The strains were mixed in a 1:1 ratio with a competing strain carrying a constitutive *lacZ* on the pVSV103 plasmid, and the mixed cultures were used to inoculate *E. scolopes* hatchlings with approximately 10^3^ to 10^4^ CFU/mL. At 3 hpi, the hatchlings were washed and transferred to 40 mL of FSIO. Water was changed at 24 hpi, and hatchlings were euthanized at 48 hpi by storage at −80°C. Euthanized hatchlings were homogenized and plated on LBS/X-Gal agar. The competitive index of the strains was measured by calculating the blue/white colony ratios as previously described (25, 29).

### Squid aggregation assays.

Strains were grown overnight in liquid LBS at 25°C on a rotator. *E. scolopes* hatchlings were inoculated with approximately 10^5^ to 10^8^ CFU/mL of bacteria. At 3 hpi, squid were anesthetized in 2% ethanol in FSIO and immediately dissected and imaged as described below, or they were fixed in 4% paraformaldehyde in 1× mPBS (50 mM phosphate buffer, 0.45 M NaCl [pH 7.4]) for approximately 48 h. Fixed hatchlings were washed four times in 1× mPBS, dissected, and imaged using a Zeiss Axio Zoom.v16 large-field 561 fluorescent stereo microscope. The aggregate area was selected, and fluorescence levels were measured using the Zen Blue Software polygon tool. Note that the analyses were conducted with all data as well as solely with the inocula in the 10^6^ to 10^7^ range, and the same results were observed, so we included all of the data in the manuscript.

### Data analysis.

Congo red binding was quantified using ImageJ by subtracting the WT gray value from the mutant gray value and multiplying the value by −1. The fluorescence levels of strains in liquid culture were measured by using a BioTek Synergy Neo2 plate reader. The fluorescence levels of strains spotted on agar plates and bacterial aggregates in the squid were measured by using Zen Blue Software. GraphPad Prism was used to generate graphs and to conduct statistical analyses. Graphs were further refined in Adobe Illustrator.
