# Supplementary material for: High Levels of Cyclic Diguanylate Interfere with Beneficial Bacterial Colonization
Source: mBio. 2022 Aug 2;13(4):e01671-22. doi: 10.1128/mbio.01671-22 (PMC9426504; doi:10.1128/mbio.01671-22)
Supplement: FIG S1 [file mbio.01671-22-s0001.pdf]

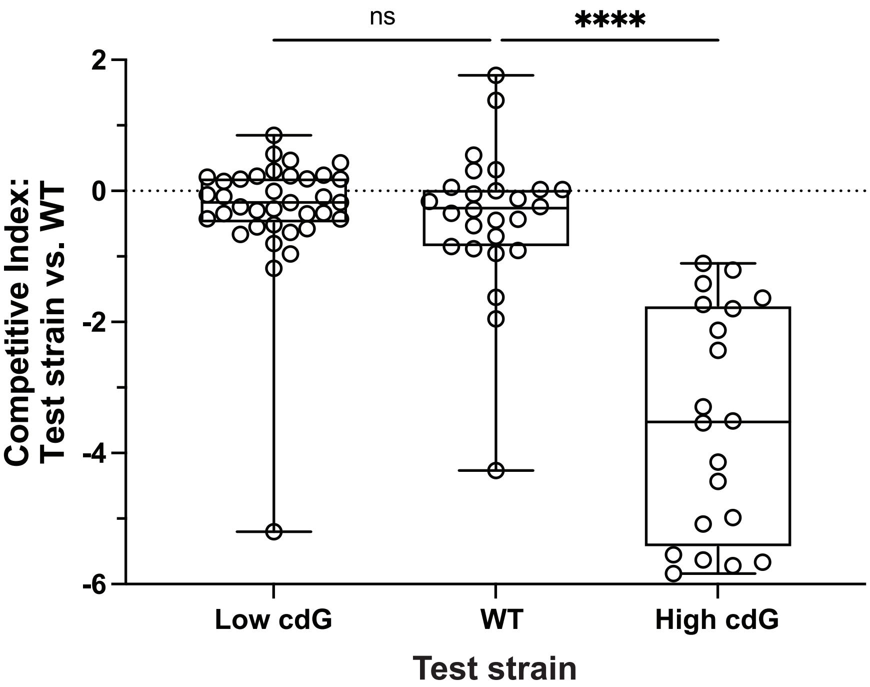

FIG S1 Low levels of c-di-GMP do not inhibit host colonization. Quantification of squid competitive colonization index at 48 hpi by indicated *V. fischeri* strains. Competitive index represents $\log_{10}$((test strain/WT)$_{output}$ / (test strain/WT)$_{input}$). Box-and-whisker plots represent minimum, 25th percentile, median, 75th percentile, and maximum. Sample sizes from left to right are 36, 26, and 20 squid. Kruskal-Wallis test was performed for statistical analysis; ns = not significant, ****$p < 0.0001$.
