# Supplementary material for: High Levels of Cyclic Diguanylate Interfere with Beneficial Bacterial Colonization
Source: mBio. 2022 Aug 2;13(4):e01671-22. doi: 10.1128/mbio.01671-22 (PMC9426504; doi:10.1128/mbio.01671-22)
Supplement: FIG S2 [file mbio.01671-22-s0002.pdf]

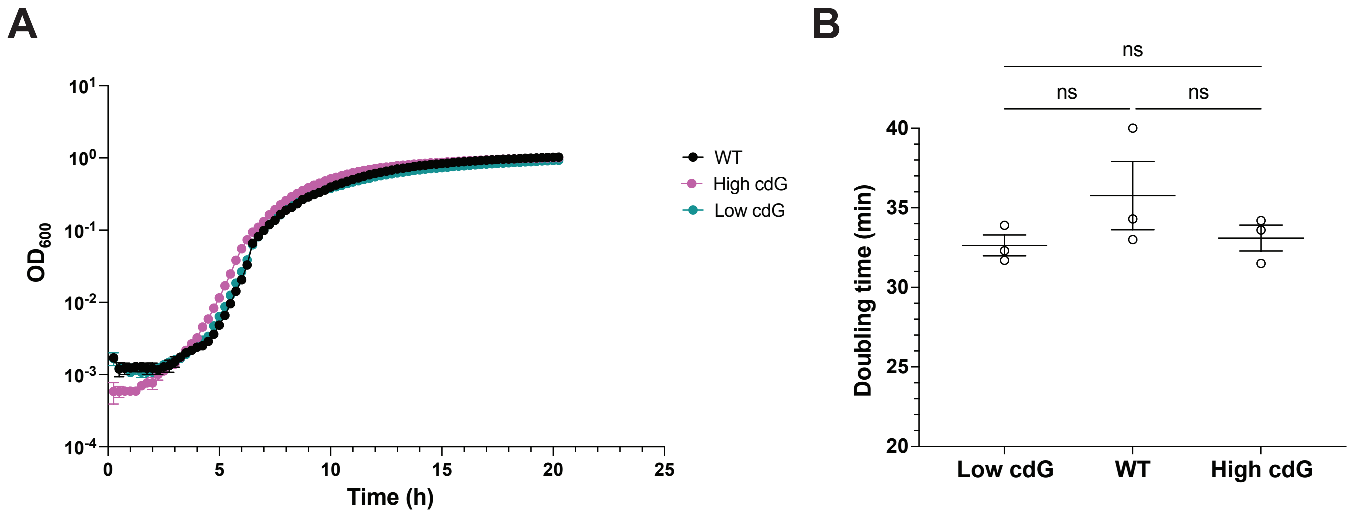

FIG S2 The High c-di-GMP *V. fischeri* strain does not have a growth defect. (A) Growth curves for *V. fischeri* and indicated mutants. For each strain, $n = 3$ biological and $n = 6$ technical replicates per biological replicate. Points represent the mean of technical replicates. Error bars represent standard error of the mean. (B) Doubling times for *V. fischeri* and indicated mutants. Average bars represent the mean of biological replicates. Points represent the mean of technical replicates. Error bars represent standard error of the mean. One-way ANOVA was used for statistical analysis; ns = not significant. Doubling time was calculated using a nonlinear regression analysis of each strain during exponential growth phase. For panels A and B, outliers were excluded from analysis.
