# Supplementary material for: High Levels of Cyclic Diguanylate Interfere with Beneficial Bacterial Colonization
Source: mBio. 2022 Aug 2;13(4):e01671-22. doi: 10.1128/mbio.01671-22 (PMC9426504; doi:10.1128/mbio.01671-22)
Supplement: FIG S3 [file mbio.01671-22-s0003.pdf]

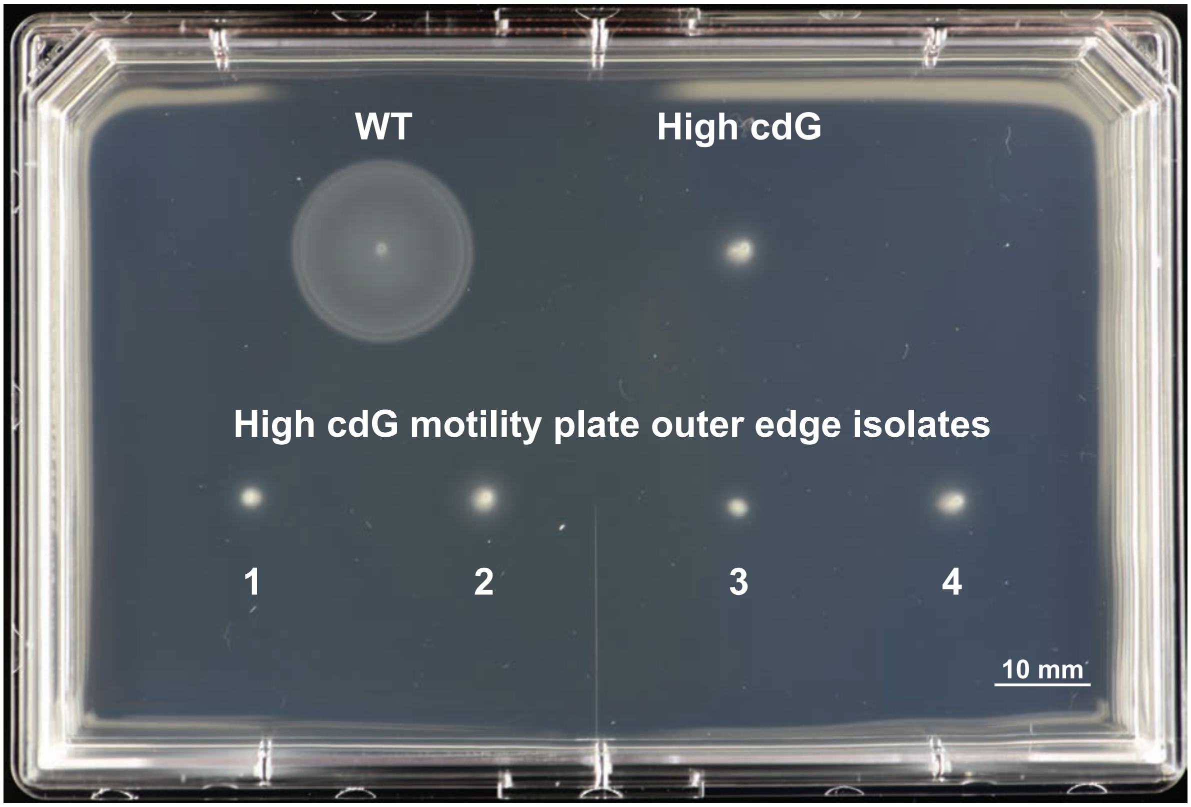

FIG S3 High cdG motile cells are not suppressor mutants. Representative image of migration through soft (0.3%) agar for *V. fischeri* and indicated strains.
