# Supplementary material for: High Levels of Cyclic Diguanylate Interfere with Beneficial Bacterial Colonization
Source: mBio. 2022 Aug 2;13(4):e01671-22. doi: 10.1128/mbio.01671-22 (PMC9426504; doi:10.1128/mbio.01671-22)
Supplement: FIG S4 [file mbio.01671-22-s0004.pdf]

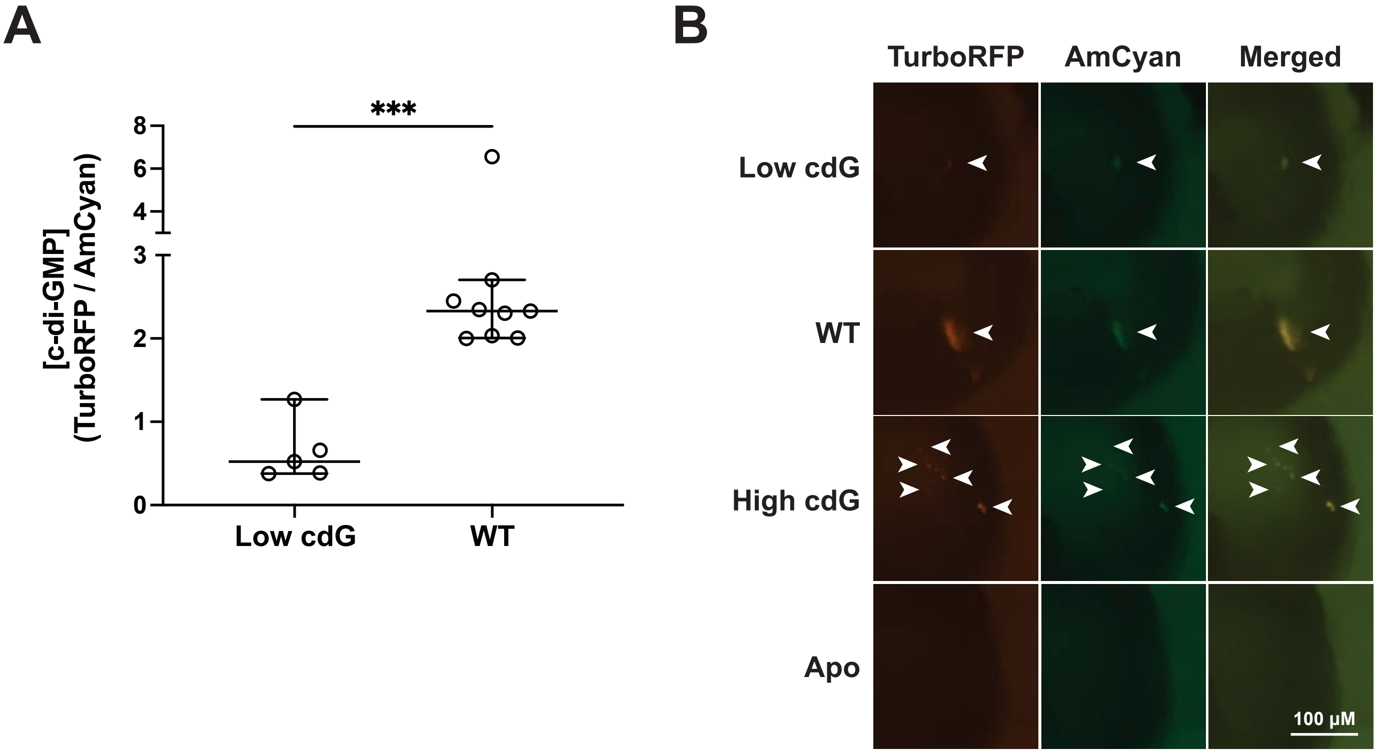

FIG S4 Multiple gene deletions alter *in vivo* c-di-GMP levels. (A) Quantification of c-di-GMP levels for indicated *V. fischeri* strains using the pFY4535 c-di-GMP reporter plasmid in aggregates within the host mucus. Samples sizes from left to right are 9 and 5 aggregates. A Mann-Whitney test was used for statistical analysis; ***$p \leq 0.01$. (B) Representative fluorescent microscopy images of squid light organs containing indicated *V. fischeri* strains carrying the pFY4535 c-di-GMP reporter plasmid. Arrows indicate location of aggregates.
