# Supplementary material for: High Levels of Cyclic Diguanylate Interfere with Beneficial Bacterial Colonization
Source: mBio. 2022 Aug 2;13(4):e01671-22. doi: 10.1128/mbio.01671-22 (PMC9426504; doi:10.1128/mbio.01671-22)
Supplement: FIG S6 [file mbio.01671-22-s0006.pdf]

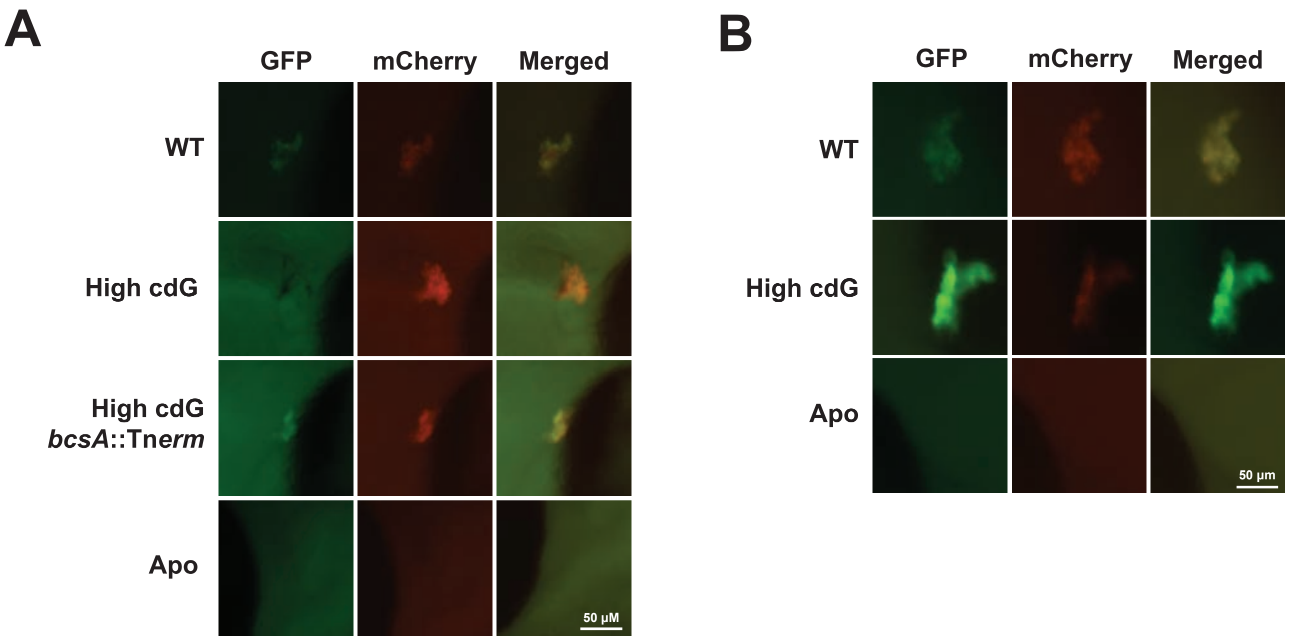


**FIG S6** High c-di-GMP-dependent Bcs expression inhibits *syp* expression in bacterial aggregates within the host mucus. (A) Representative fluorescent microscopy images of squid light organs containing indicated *V. fischeri* strains carrying the pM1422 *sypA*'-*gfp*$^+$ transcriptional reporter plasmid. (A) Representative fluorescent microscopy images of squid light organs containing indicated *V. fischeri* strains carrying the pRYI063 *bcsQ*'-*gfp*$^+$ transcriptional reporter plasmid.
